# Case Report: Transformation of natural killer-cell large granular lymphocytic leukemia to aggressive natural killer cell leukemia

**DOI:** 10.3389/fonc.2025.1648711

**Published:** 2025-08-29

**Authors:** Wen Wang, Ke Lu, Lijie Xing, Zengjun Li

**Affiliations:** Department of Lymphoma, Shandong Cancer Hospital and Institute, Shandong First Medical University and Shandong Academy of Medical Sciences, Jinan, Shandong, China

**Keywords:** NK-LGLL, ANKL, LGLL, treatment, diagnosis

## Abstract

Large Granular Lymphocytic Leukemia (LGLL) is a rare clonal proliferative disorder of cytotoxic T lymphocytes (CTL) and natural killer (NK) cells, characterized by persistent expansion of large granular lymphocytes (LGLs) in peripheral blood for over six months. According to the 2022 World Health Organization (WHO) classification, LGLL is categorized into T-LGLL, NK-large granular lymphocytic leukemia (NK-LGLL). Aggressive natural killer cell leukemia (ANKL), as a Mature T-cell and NK-cell leukemia independent of LGLL, also has morphologic characteristics of large granular lymphocytes. This report describes a rare case of NK-LGLL transforming into ANKL. This case highlights the necessity of differential diagnosis in LGLL and suggests that proteasome inhibitors combined with immune checkpoint inhibitors may represent a promising therapeutic strategy for ANKL.

## Introduction

LGLs are defined morphologically as large lymphoid cells with round or renal nuclei and abundant cytoplasm containing azurophilic granules with a diameter of 15 to 18 μm (approximately twice that of typical lymphocytes). In the physiological state, LGLs typically constitute 10 to 15% of peripheral white blood cells. LGLL is a clinically heterogeneous disease characterized by persistent clonal expansion of LGLL (≥2×10^9^/L) in peripheral blood for more than 6 months without clear etiology. LGLL can be divided into T-LGLL and NK-LGLL according to phenotype, accounting for 85% and 15%, respectively, and share common pathophysiology and manifestations. LGLL is an indolent disease, with 10-year overall survival (OS) of more than 70% for T-LGLL and 65% for NK-LGLL. LGLL accounts for 2-5% and 5-6% of chronic lymphoproliferative disorders in Western and Asian populations (1, 2), respectively, with a median age of 60 to 65 years (<20% of cases < 50 years) (3, 4). Approximately one-third of LGLL patients remain asymptomatic, diagnosed incidentally through routine hematologic evaluation, while symptomatic manifestations primarily include cytopenias (e.g., neutropenia-related infections, anemia-induced fatigue), splenomegaly, or autoimmune phenomena (5). Notably, reactive LGL expansions demonstrate overlapping clinicopathological features with LGLL but exhibit stronger associations with post-splenectomy status, viral infections, or post-transplant immunological contexts (6, 7). ANKL is a rare and aggressive hematologic malignancy that accounts for 0.1% of lymphoid neoplasms (8). It most commonly affects young patients between the ages of 20 and 30 years, and is mainly prevalent in East Asian countries. The median OS is < 2 months (9).

## Case presentation

A 65-year-old male initially presented with abdominal discomfort. In May 2023, he received a diagnosis of LGLL at a local hospital and was subsequently admitted to our hematology department in August 2023. Laboratory investigations revealed a white blood cell count of 1.22×10^9^/L, a neutrophil count of 0.51×10^9^/L, a red blood cell count of 2.56×10¹²/L, hemoglobin level of 75 g/L, platelet count of 56×10^9^/L, triglycerides level of 3.94 mmol/L, serum ferritin concentration of 1951.00 ng/ml, and lactate dehydrogenase level of 728 U/L, findings consistent with Hemophagocytic Lymphohistiocytosis (HLH). Karyotype analysis and EBV-DNA quantification revealed no abnormalities. Bone marrow morphology, assessed via Wright-Giemsa staining, demonstrated 16% immature abnormal lymphocytes characterized by larger cell bodies, reduced cytoplasm, gray-blue staining, irregular cytoplasmic edges, and occasional hemophagocytes, consistent with LGL features. Flow cytometry of the bone marrow indicated strong CD56+, CD16±, CD3-, with 31.8% of CD56+CD3- cells expressing CD16+, suggestive of a NK cell phenotype. Differentiation between NK-LGLL and ANKL is primarily based on clinical presentation, with ANKL being highly aggressive and associated with a median overall survival of less than 2 months. Given the absence of specific symptoms and the lack of b symptoms, such as fever, from the onset of symptoms to the initiation of treatment, the clinical presentation was inconsistent with ANKL. Consequently, the final diagnosis was NK-LGLL with HLH. Due to the lack of standardized treatment for NK-LGLL, a TPM regimen (Thalidomide 100 mg qd + Prednisone 35 mg every other day + Methotrexate 16.6 mg qw) was administered. The NCT04453345 clinical study demonstrated a better overall response rate (ORR) and complete response (CR) rate with fewer adverse effects compared with previous data on immunosuppressive therapy (2). According to efficacy evaluation criteria, CR was defined as absolute neutrophil count (ANC) > 1.5 × 10^9^/L, hemoglobin (HGB) level >11 g/dL, and platelet count (PLT) > 100 × 10^9^/L; hematological Partial Response (PR) was defined as an improvement in the blood ANC > 0.5 × 10^9/^L, an increase in the HGB level of >1 g/dL, a PLT > 50 × 10^9^/L and the absence of transfusion requirement. Progressive disease (PD) was defined as a worsening of hematological parameters (a decrease in the HGB level of 2 g/dL or a HGB level less than 10 g/dL, a decrease in the ANC of 0.5 × 10^9^/L or an ANC less than 1.0 × 10^9^/L, a decrease in the PLT of 20 × 10^9/^L in PLT and a PLT less than 100 × 10^9^/L, or transfusion requirement) or findings of organomegaly, such as hepatosplenomegaly, are detected in patients previously achieving PR/CR. CR was achieved in September 2023, with sustained remission observed in December 2023.

**Figure 1:**
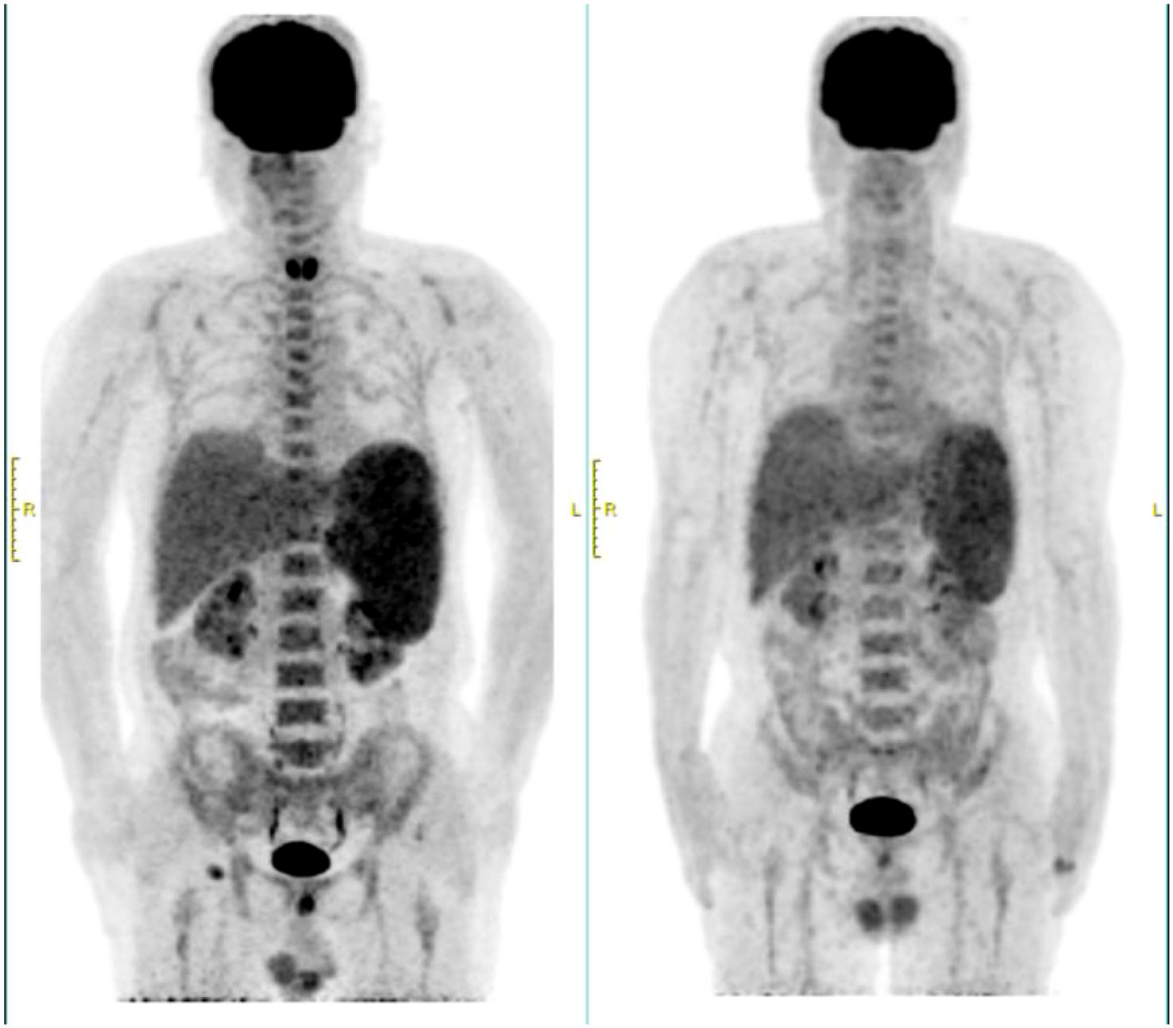
PEC/CT at recurrence and initial diagnosis. The left side was at recurrence in March 2024, and the right side was at initial diagnosis in August 2023.

In March 2024, the patient experienced recurrent fever up to 39°C and significant fatigue. Despite anti-infection treatments at a local hospital, symptoms persisted, leading to readmission. Laboratory findings included a white blood cell count of 2.72×10^9^/L, neutrophil count of 2.03×10^9^/L, hemoglobin of 97 g/L, and platelet count of 103×10^9^/L. Compared to the initial diagnosis PET-CT, the current scan reveals hepatosplenomegaly with increased metabolic activity, diffusely homogeneous hypermetabolism throughout the skeletal system, and enlarged hypermetabolic lymph nodes in the right inguinal region (**Figure 1**). The percentage of abnormal NK cells in bone marrow was 25.6%. HE and PAS staining showed active bone marrow hyperplasia (about 50%), atypical lymphocytes aggregation accounted for 10% of nuclear cells, reduced cytoplasm, irregular nuclei, fine chromatin, and visible nucleoli. The tumor cells were positive for CD2, CD56, TIA-1 and Granzyme B, but negative for CD3 and CD5. Karyotype analysis revealed 44, X, -Y, i (7) (q1), del (9) (p21), der (10, 11) (q10; q10), inv (12) (p11.2q25) [3]/46, XY [27]. EBV-DNA quantification was normal. Serum ferritin: 1396ng/ml. Combined with the clinical manifestations of persistent fever, hepatosplenomegaly, and significantly elevated ferritin, the diagnosis was revised to EBV-negative ANKL.

**Figure 2:**
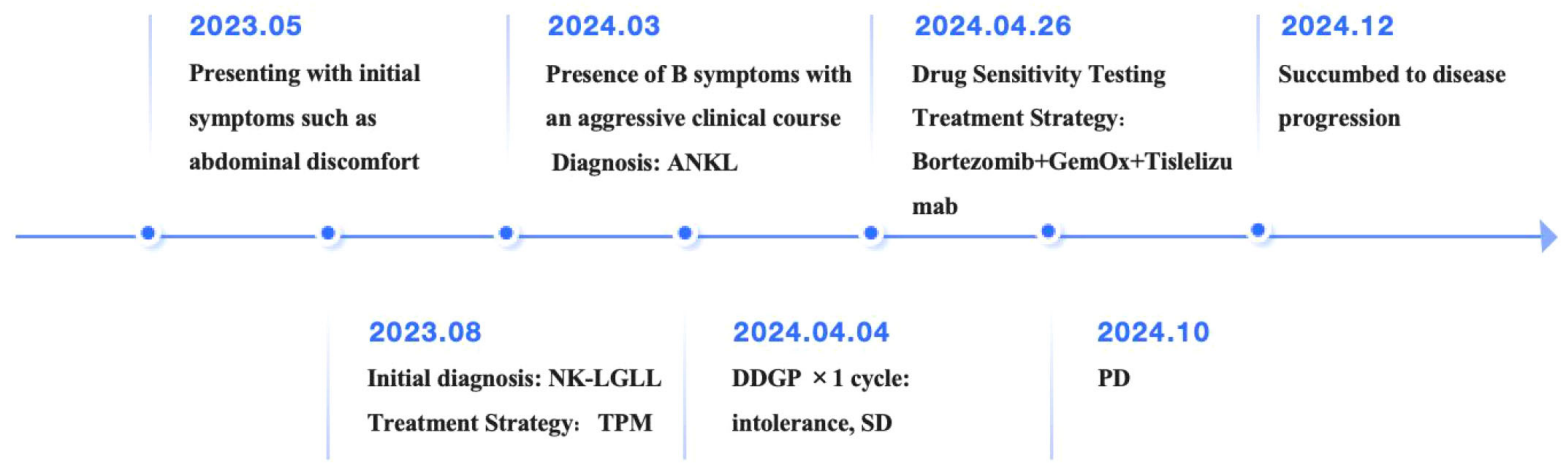
Visualization of the patient’s clinical course.

In terms of treatment strategy, the DDGP regimen, which includes Gemcitabine, was employed. After one cycle of therapy, the patient’s response was evaluated as stable disease (SD). However, the patient exhibited poor tolerance to this regimen, and recurrent fever symptoms did not show significant improvement. To explore more effective treatment options, ex vivo drug sensitivity testing was performed using primary tumor cells isolated from the patient’s bone marrow, revealing high sensitivity to both bortezomib and carfilzomib. Additionally, the patient demonstrated high sensitivity to chemotherapy regimens GDP (Gemcitabine + Dexamethasone + Cisplatin), VTD (Bortezomib + Thalidomide + Dexamethasone), ICE (Ifosfamide + Carboplatin + Etoposide), and FLAG (Fludarabine + Cytarabine (Ara-C) + G-CSF) (**Supplementary Figure S1**). Bortezomib, a proteasome inhibitor, is capable of downregulating the NF-κB signaling pathway. Previous studies have indicated that the NF-κB pathway remains persistently active in LGLL and inhibits apoptosis independently of STAT3 activation (10, 12). Proteasome inhibitors, including Bortezomib and Ixazomib, have been shown to suppress the NF-κB pathway, thereby reducing the proliferation of LGL leukemia cell lines and primary patient cells, and promoting apoptosis (13). Furthermore, immunotherapy is considered a potential treatment strategy for ANKL. Previous studies have shown that Integration of anti-PD-1 antibody into chemotherapeutic regimens improved the outcome of aggressive NK cells leukemia (14). Studies have also demonstrated overexpression of PD-L1 in EBV-negative ANKL patients, suggesting a viable therapeutic target for this rare disease (11). Based on the drug sensitivity results, prior research findings, and consideration of the patient’s tolerance (Unable to tolerate the GDP regimen, GemOx regimen was empirically administered), a personalized treatment regimen combining GemOx (Gemcitabine+ Oxaliplatin), Tislelizumab (an anti-programmed cell death protein-1 antibody), and Bortezomib was devised. Fortunately, after one cycle of treatment, CT scans revealed no abnormal lymphadenopathy, and no abnormal NK cells were detected in the peripheral blood. After two cycles, the patient’s fever symptoms were alleviated, and both peripheral blood and bone marrow flow cytometry showed no detectable phenotypically abnormal NK cells (below the detection limit: 0.006%). MRD remained undetectable until October 2024, when 0.80% of peripheral blood nuclear cells showed an abnormal NK-cell phenotype, indicating disease progression (**Supplementary Figure S2**). The response thus lasted approximately 5 months following the achievement of CR in April 2024.The patient died of disease progression in December of the same year. **Figure 2** is a visualization illustrating the clinical course of the patient.

## Discussion

In this case, we should first focus on the differential diagnosis between NK-LGLL and ANKL. **Table 1** lists the differential diagnosis between NK-LGLL and ANKL. Accurate diagnosis mainly relies on cytology, immunophenotype and clonal evidence as well as clinical manifestations (15). In terms of cytological characteristics, both NK-LGLL and ANKL have the characteristics of large granular lymphocytes in peripheral blood (16). The typical characteristics are that the absolute count of LGL exceeds 0.5× 10^9^/L, the cell size is large (diameter 15-18µm), and the granules are nitrogen-philic. In terms of immunophenotype, both of them exhibit the phenotype of s CD3-, CD7-, CD4-, CD16+, CD56+ (17). CD57 is variably expressed (18). Specifically, NK-LGLL is mostly CD56-/+ dim, CD94 + bright, CD16+, CD57-/+ dim. ANKL is mostly CD56 + bright, CD94 + bright, CD26+, CD16-/+ dim, CD57- (19). Both express antigens related to NK, such as CD94. Both of them are positive in cytotoxic proteins (TIA-1, granzyme B and granzyme M). In terms of clonal assessment, both have restricted expressions of NK cell receptors, killer cell immunoglobulin-like receptor (KIRs) and NKG2/CD159. In summary, the flow cytometric phenotypes of NK-LGLL and ANKL are extremely similar. According to the 5th edition of WHO, the minimum diagnostic criteria for ANKL include: 1. Presentation with fever, constitutional symptoms and a leukaemic blood picture. 2. Systemic (multiorgan) involvement by neoplastic lymphoid cells with an NK cell immunophenotype. 3.Absence of T cell receptor protein expression and/or clonal TR rearrangement. Therefore, systemic symptoms are essential for the differential diagnosis of ANKL and NK-LGLL (20). NK-LGLL is usually negative for EBV, has a slow disease course and a good prognosis (21). Patients with LGLL usually do not develop B symptoms. However, some patients may experience abdominal discomfort due to enlarged organs. Splenomegaly occurs in approximately 20% to 30% of patients with LGLL, whereas hepatomegaly is present in approximately 10% of cases (4). Conversely, ANKL is often accompanied by EBV infection (only 10% are EBV negative), with extremely rapid disease progression (22, 23). Most patients often present with hemophagocytic syndrome and disseminated intravascular coagulation (DIC), mainly involving the bone marrow, peripheral blood, liver and spleen. The median OS < 2 months. The most important differential diagnosis between the two lies in their clinical manifestations. This case indicates that NK-LGLL can be transformed into ANKL. Therefore, in clinical practice, if the patient’s condition progresses rapidly, we must be vigilant about whether it will be transformed into ANKL.

**Table 1:** Differential diagnosis between NK-LGLL and ANKL.

Elements of identification	NK-LGLL	ANKL
Cytology	The cells are relatively large in volume (with a diameter of 15-18 μ m), have a moderate to large amount of cytoplasm, and contain acanthopanax granules.	
Immunophenotype	CD3−, CD7-, TIA-1+, granzyme B +, granzyme M+	
	CD8+(uniform positivity), CD56-/+ dim, CD94 + bright, CD16+	CD8-, CD56 + bright, CD94 + bright, CD26+, CD16-/+ dim
Clonal evidence	NK cell receptor-restricted expression: killer cell immunoglobulin-like receptor (KIRs) and NKG2/CD159	
Clinical manifestations	usually negative for EBV, Indolent disease course, Favorable prognosis with rare progression to ANKL	90% EBV+, often accompanied by HLH and DIC, median OS < 2 months

Currently, there is no definitive standard therapy for LGLL. The pathogenesis of NK-LGLL remains unclear, though most studies suggest that exogenous antigenic stimulation (e.g., various viruses) and autoimmune dysfunction ultimately lead to abnormal NK cell proliferation. Treatment strategies for NK-LGLL are similar to those for T-LGLL, involving observation for asymptomatic patients and immunosuppressive therapy for those with symptomatic cytopenias or associated autoimmune conditions. Common immunosuppressants include Methotrexate, Cyclosporine, and Cyclophosphamide (24). Previous studies of immunosuppressive therapy for NK-LGLL have been mostly retrospective and of limited efficacy. In these studies, overall response rates (ORR) were similar for the three immunosuppressants, ranging from 21% to 85% (median, 50%), while complete remission rates were relatively low, ranging from 2-31% for methotrexate, 29-100% for cyclophosphamide, and 4-19% for cyclosporine (25–28). A multicenter, prospective phase II trial (NCT04453345) showed that TPM (thalidomide + prednisone + methotrexate) regimen was significantly effective and well tolerated in patients with LGLL. The CR rate was 75.0%, and the overall response rate (ORR) was 90.4%, which was the highest response rate reported so far. With this treatment, hemoglobin levels normalized in 83% of patients and neutrophil levels normalized in 71.3%. The median follow-up time was 29 months, the median progression-free survival (PFS) was 40 months, and the median duration of response (DoR) was 39 months (2).

For the treatment of ANKL, patients with ANKL are insensitive to conventional chemotherapy regimens, based on the fact that anthracycline-based chemotherapy regimens are usually ineffective, and L-asparaginase-based combination chemotherapy regimens may be considered such as SMILE (Dexamethasone,Methotrexate, Ifosfamide,L-asparaginase,Etoposide) (29),AspaMetDex(L-asparaginase,Methotrexate, Dexamethasone),VIDL (Etoposide, Ifosfamide, Dexamethasone, L-asparaginase),P-Gemox (Gemcitabine, Oxaliplatin, Proteasome inhibitor) (30). However, complete remission is achieved in less than 20% of patients, often requiring hematopoietic stem cell transplantation (HSCT) to improve outcomes (31, 32). For this patient, we initially chose the DDGP (dexamethasone, cisplatin, gemcitabine and pembrolase) regimen, which showed better efficacy and safety than the SMILE regimen in the late-stage ENKL trial (NCT01501149) (33). Personalized therapy based on drug sensitivity testing led to successful remission, emphasizing the importance of tailored treatment approaches in refractory or relapsed cases.

Additionally, novel agents are being explored for NK-LGLL treatment. The histone deacetylase inhibitor vorinostat shows significant inhibitory effects on NK cell line proliferation possibly through inhibition of the JAK-STAT pathway (34). Drug sensitivity assays have identified JAK inhibitors like Ruxolitinib and BCL2 inhibitors such as Venetoclax as highly effective against malignant NK cell lines (35). While many of these agents are approved for other hematologic malignancies, their clinical efficacy in ANKL requires further validation.

Finally, in a disease with the potential for aggressive transformation, we should explore the molecular mechanisms of transformation and identify genetic and immune-system alterations that contribute to disease progression. Understanding these mechanisms will be critical for developing targeted therapies and improving patient outcomes (36). Therefore, the molecular transformation mechanism of NK-LGLL to ANKL should be emphasized in the future.

## Conclusion

This case illustrates the potential transformation of NK-LGLL into ANKL, emphasizing the necessity for continuous monitoring and accurate differential diagnosis in patients with LGLL. The successful implementation of personalized treatment strategies, informed by drug sensitivity testing, highlights the effectiveness of tailored therapeutic approaches for managing refractory or aggressive disease manifestations. Furthermore, the promising response to the combination of proteasome inhibitors and immune checkpoint inhibitors suggests a viable and innovative treatment avenue for ANKL patients. Future research should focus on validating these personalized treatment protocols and exploring novel agents that target specific molecular pathways involved in NK cell malignancies to improve treatment outcomes and patient prognosis.

## Data Availability

The original contributions presented in the study are included in the article/**Supplementary Material**. Further inquiries can be directed to the corresponding authors.
