# Supplementary material for: Case Report: Transformation of natural killer-cell large granular lymphocytic leukemia to aggressive natural killer cell leukemia
Source: Front Oncol. 2025 Aug 29;15:1648711. doi: 10.3389/fonc.2025.1648711 (PMC12425761; doi:10.3389/fonc.2025.1648711)
Supplement: Supplementary file 1 [file DataSheet1.docx]

Fig.S1 Drug sensitivity test results of bone marrow tumor cells of patients.

| **HDS Test Results Summary** | | | | | |
| --- | --- | --- | --- | --- | --- |
|  | | **Highly sensitive** | **Moderately sensitive** | **Low-level sensitive** | **Not sensitive** |
| **Cytotoxic drugs** | **Alkylating agents** | —— | —— | —— | Dacarbazine, bendamustine, Chlorambucil, busulfan, Cyclophosphamide, Lomustine, ifosfamide, L-phenylalanine nitrogen mustard, Carmustine, Procarbazine |
|  | **Antimetabolites** | —— | Cytarabine(3000 mg/ m^2^、2000 mg/ m^2^) , fludarabine and gemcitabine | Hydroxyurea(60mg/kg), decitabine, 5-fluorouracil (750 mg) | Cytarabine (100 mg/ m^2^), Azacitidine, Hydroxyurea (20 mg/kg), Methotrexate, Clofarabine,  Cladribine, 6-mercaptopurine, 5-fluorouracil  (250 mg) |
|  | **Anti-microtubule agents** | —— | Taxol, docetaxel | Vinblastine, vindesine | Vinorelbine, vincristine |
|  | **Topoisomerase inhibitors** | —— | Hydroxycamptothecin, irinotecan | Teniposide (60 mg/m^2^), topotecan, etoposide | Teniposide (30 mg/m^2^) |
|  | **Antibiotics** | —— | Epirubicin, daunorubicin (90 mg/m^2^) | Acarubicin (1mg/kg), mitoxantrone (14mg/m^2^), Daunorubicin  (60 mg/m^2^, 40 mg/m^2^), doxorubicin, pirarubicin, bleomycin | Acarubicin (0.4mg/kg), mitoxantrone (8 mg/m^2^), idarubicin mitomycin C |
|  | **Antiviral drugs** | —— | —— | Adefovir dipivoxil | —— |
|  | **Others** | —— | Homoharringtonine (4mg), deferoxamine, arsenic trioxide | Homoharringtonine (1mg), amsacrine | Cisplatin, bexarotene, oxaliplatin, deferasirox, carboplatin, all-trans retinoic acid |
| **Supportive Care Medications** | **Hormonal Agents** | —— | —— | Methylprednisolone, hydrocortisone, prednisone, dexamethasone | —— |
|  | **Immunomodulatory Agents** | —— | —— | Pomalidomide, mycophenolate mofetil | Thalidomide, lenalidomide |
| **Targeted Drugs** | | Bortezomib,  Carfilzomib | —— | Ponatinib | Dasatinib, chidamide, Ruxolitinib, Venetoclax, Midostaurin, nilotinib, bosutinib, imatinib, ixazomib, ibrutinib |
| **Chemotherapy Regimens** | | VTD, ICE, GDP, FLAG | HDMTX+L-ASP, VDLP, VDLD, VDCLP, TA ,Selinexor+Dexamethasone, MOAP, MO ACD, IOLP, IOAP, Hyper-CVAD(B), HOAP, HD-DA, HAD, HAA, HA, ESHAP, DOMP, DOLP, DHAP, DAT, DAC, DA-EPOCH, DA, COP, COATD, CLAG, CHOP, CHOEP, CAM, CAG | Mm, MINE, ME, MA, IAE, IA, Hyper-CVAD(A), DCAG, DAE, A BVD | 6-MP+MTX |

Fig.S2

A


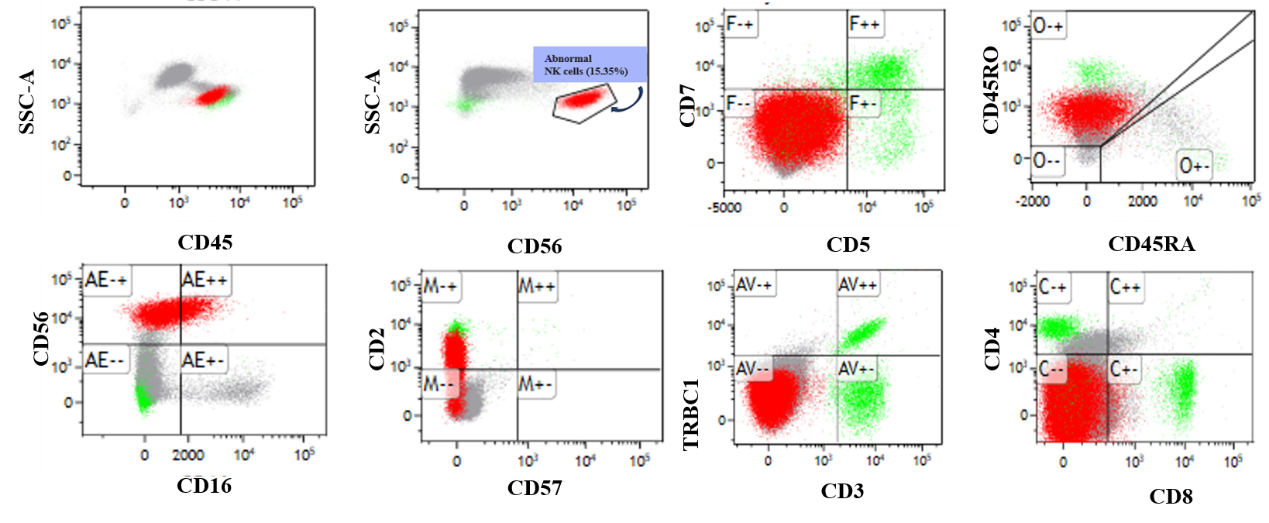


B


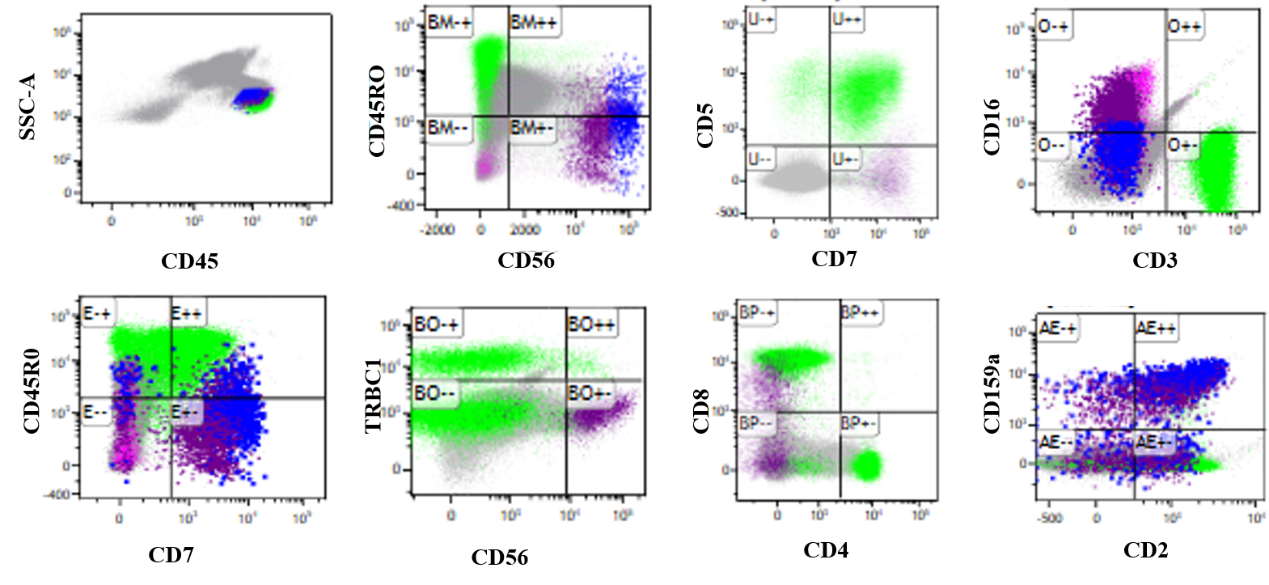


C


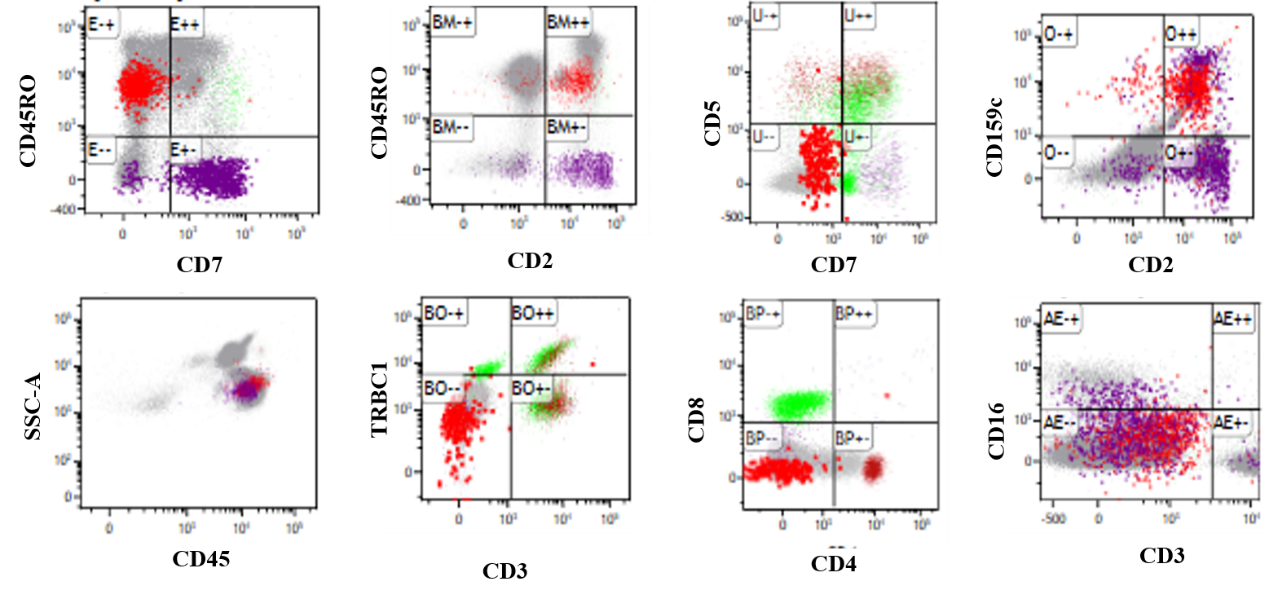


(A) is the flow cytometry of the patient at relapse in March 2024, 15.35% of NK cells with abnormal phenotype (CD16 mostly -, CD45RO+, and CD7 -) were detected in the peripheral blood. No normal NK cells were detected; (B) showed that NK cells with abnormal phenotype were not detected in peripheral blood of patients after one cycle of Bortezomib+GemOx+Tislelizumab treatment. Normal NK cells accounted for 0.80% of the nuclear cell count; (C) is 0.80% NK cells with abnormal phenotype (CD16-, CD7-, CD159 shows monoclonal expression) detected in peripheral blood at the time of second progression in October 2024. Normal NK cells accounted for 1.5% of the nuclear cell count. The red population represents abnormal NK cells (Diagnosis of aberrant NK cells requires combined phenotypes (e.g., CD56briCD7-CD45RO+) with morphological correlates (e.g., cell enlargement). the mere presence of a CD7-CD45RO+subpopulation does not confirm a neoplastic origin unless accompanied by strong CD56 expression and enlarged cell morphology.); The green population represents T cells; The blue and purple populations represent normal NK cells. NK1 cells (CD56+CD16dim) are shown in blue, and NK2 cells (CD56+CD16+) are shown in purple. The normal NK cell phenotype is defined as: CD3−, CD56+, with variable (non-uniform) expression of CD7, CD16, CD45RO, and CD2.
